# Self Complacency

**Published:** 1859-12

**Authors:** 


### SELF COMPLACENCY.

DURING the last week of October, eighteen hundred and fifty-nine, of all the persons who died in Boston more than one third were the victims of consumption, and yet, the Bostonians account themselves the most learned, refined, and cultivated, in all this wide land. Few Greenlanders die of the terrible malady ; not two per cent. of the deaths in the City of Mexico are of consumption, so that this fell disease does not appear to be the peculiar child of heat or cold or latitude, it is founded in unwise habits of life; unwisdom in eating, in drinking, in recreations, in exposures, in the daily habits of business and social life. The Bostonians seem to have made as unfortunate a use of their self-claimed intelligence in the preservation of the physical health of their city, as they have in their religions. It is from Boston that the keenest shafts against the Christian religion have been sped ; orthodoxy, the faith of the fathers of the olden time, is the target of renegade sons, and save the mark, of daughters too. When a minister's daughter and a minister's son so write that the uncircumcised come up, and catching them gladly by the hand exclaim, with beaming eyes, "Brother!" may we not inquire whether self complacency is not a physical and moral nuisance which ought to be abated "*vi et armis.*"

